# High prevalence of overweight/obesity and central obesity among women in a rural district of Nepal in 2012–2013: a population-based study

**DOI:** 10.3389/fpubh.2025.1455940

**Published:** 2025-02-06

**Authors:** Chandra Yogal, Astrid Kamilla Stunes, Sunila Shakya, Biraj Karmarcharya, Rajendra Koju, Mats P. Mosti, Miriam K. Gustafsson, Bjørn Olav Åsvold, Berit Schei, Unni Syversen

**Affiliations:** ^1^Department of Clinical and Molecular Medicine, Faculty of Medicine and Health Science, Norwegian University of Science and Technology, Trondheim, Norway; ^2^Department of Community Program, Kathmandu University School of Medical Science, Dhulikhel Hospital, Kathmandu University Hospital, Dhulikhel, Nepal; ^3^Center for Oral Health Services and Research, Mid-Norway (TkMidt), Trondheim, Norway; ^4^Department of Gynecology and Obstetrics, Kathmandu University School of Medical Sciences, Dhulikhel Hospital, Kathmandu University Hospital, Dhulikhel, Nepal; ^5^Department of Internal Medicine, Dhulikhel Hospital, Kathmandu University Hospital, Dhulikhel, Nepal; ^6^Department of Research and Development, Clinic of Substance Use and Addiction Medicine, St. Olavs University Hospital, Trondheim, Norway; ^7^Regional Education Center, Helse Midt-Norge, Trondheim, Norway; ^8^HUNT Center for Molecular and Clinical Epidemiology, Department of Public Health and Nursing, Faculty of Medicine and Health Science, Norwegian University of Science and Technology, Trondheim, Norway; ^9^Department of Endocrinology, Clinic of Medicine, St. Olavs University Hospital, Trondheim, Norway; ^10^Department of Public Health and Nursing, Faculty of Medicine and Health Science, Norwegian University of Science and Technology, Trondheim, Norway

**Keywords:** overweight/obesity, central obesity, instant noodles, milk intake, women, rural Nepal, prevalence

## Abstract

**Background:**

The prevalence of overweight is rapidly escalating, especially in South-Asia. We aimed to estimate the prevalence of overweight/obesity and central obesity, and associated risk factors among women in a rural setting of Nepal.

**Methods:**

A cross-sectional study addressing reproductive health and non-communicable diseases (NCDs) was conducted 2012–2013 in a rural district of Nepal. Married, non-pregnant women ≥15 years of age were included. Height, weight, and waist circumference (WC) were measured, and body mass index (BMI) calculated. WHO cut-offs for Asians were used to assess the prevalence of overweight (23.0–27.4 kg/m^2^), obesity (≥ 27.5 kg/m^2^) and central obesity (WC ≥ 80 cm). Data concerning socioeconomic and lifestyle factors were collected.

**Results:**

Altogether, 1,391 women 43.1 ± 14.4 years of age were included. The majority belonged to the Adhivasi/Janajati ethnicity, were uneducated and worked in agriculture. Altogether, 30.5% were overweight, 12.0% obese, and 34.2% centrally obese; 25.7% were both overweight/obese and centrally obese. Underweight (< 18.5 kg/m^2^) was observed in 9.6%. Among women with normal weight or underweight, 14.9 and 15.3% had central obesity, respectively. Hypertension was observed in 13.4% and was associated with both overweight/obesity and central obesity. Instant noodle intake ≥2 times weekly was associated with increased prevalence of central obesity and overweight/obesity.

**Conclusion:**

We observed a high prevalence of overweight/obesity and central obesity among women in a rural district of Nepal, which entails an increased risk of metabolic complications and NCDs. Our findings underscore the need for public health programs addressing nutritional patterns and physical activity to prevent obesity.

## Introduction

The prevalence of overweight and obesity has doubled since 1980, affecting nearly a third of the population worldwide (1). The prevalence is now rapidly escalating in low- and middle-income countries (2). The most rapid rise in obesity is seen in South Asian countries like Maldives, Bhutan, Myanmar, Nepal, and Bangladesh (3). This rise is linked among others to dietary changes, increase in sedentary lifestyle, environmental contaminants, and epigenetic influences (4). Notably, the prevalence is somewhat higher among women than in their male counterparts (5, 6).

Nepal has witnessed a rapid change in social determinants, such as urbanization, nutritional transition, and economics (7). This is also accompanied by a dramatic rise in overweight and obesity, as demonstrated by the 2016 Nepal demographic and health survey (NDHS), including a total of 13,542 adults >18 years, of whom 61% were residing in urban areas (8). The overall prevalence of overweight/obesity using cut-offs for Asians (BMI ≥ 23.0 kg/m^2^) was 31.2%. When stratifying for sex, a higher prevalence of overweight or obesity was observed among women than men (8). Women of reproductive age are even more susceptible to overweight or obesity (9). Between 1996 and 2016, overweight/obesity (BMI ≥ 25.0 kg/m^2^) and obesity (BMI ≥ 30.0 kg/m^2^) increased from 1.8 to 19.7% and 0.2 to 4.1%, respectively, among non-pregnant Nepalese women (15–49 years) using cut-offs for the general population (10). In the same time period, the prevalence of underweight decreased from 24 to 17% (11). Socioeconomic factors, older age, female sex, married, education, watching television and high income have been shown to be positively associated with overweight/obesity prevalence in Nepal (10, 12–14).

The rise in obesity prevalence represents a major challenge as it is associated with increased risk of non-communicable diseases (NCDs), such as type 2 diabetes (T2D), hypertension and cardiovascular diseases (CVDs) (15, 16). NCDs are the main causes of death globally, accounting for 71% of deaths every year (17). Vasan et al., found that a weight increase by 5% promoted a 20–30% rise in the incidence of hypertension (18). Men display a higher prevalence of hypertension, but after the onset of menopause, women experience a more rapid increase in the prevalence (19). Maternal overweight/obesity during pregnancy is also associated with increased risk of metabolic diseases in the offspring later in life (20). In addition to the consequences for the individuals, the obesity epidemic represents a substantial economic burden on the society (21).

BMI is the most widely used measure of overweight and obesity. In Asian populations, the prevalence of CVDs has been observed to increase continuously with BMI (22). Central adiposity as assessed by waist circumference (WC) has, however, emerged as a better predictor of cardiometabolic risk than generalized adiposity (23) and is a significant predictor of obesity-related diseases and all-cause mortality (24, 25). Notably, South Asians, in general, have higher body fat and lower skeletal muscle mass at the same or lower BMIs compared to white people. Despite being generally less obese, they are also more prone to visceral fat accumulation and excess hepatic fat than western populations, which implies an increased risk for T2D and CVDs (26). This phenotype is even more pronounced in women. Consequently, lower cut-off values for BMI have been set by WHO for Asian than for White populations based on risk for CVDs and T2D (27, 28). The cut-off for WC used to define central obesity, is the same in Asian and European women (29).

Most previous studies in Nepal have addressed overweight and obesity by BMI. Thus, there is a knowledge gap concerning the prevalence of central obesity and the distribution of central obesity among individuals with different BMI categories, particularly among women in rural Nepal. In the present study, we therefore aimed to estimate the prevalence of both central obesity and overweight/obesity assessed by WC and BMI, respectively, among women in a rural setting of Nepal.

## Materials and methods

### Study design, study site and population

This is a cross-sectional population-based study conducted during February 2012 to May 2013 in five villages within the Kavre District, Nepal, as described previously (30, 31). The outcomes were prevalence of sexually transmitted infections (30, 31), and prevalence of overweight/obesity and diabetes. According to local tradition, never married and pregnant women, should not undergo gynecological examination (31). Therefore, the inclusion criteria were that the women should be married (including divorced and widowed) and ≥ 15 years of age. Exclusion criteria were pregnancy and mental and physical illness that made it difficult to attend the study site.

In each of the five villages there is a governmental primary health center and additionally, in Bolde Fediche there is an outreach health center run by Dhulikhel Hospital. According to the District Population Profile 2012, a total of 7,379 females were living in these five villages (32). The governmental household records showed that 2,674 married women aged ≥15 years were living in the villages during the study period. Of these, 258 were pregnant, thus, 2,416 women were eligible for participation in the study. Female community health volunteers (FCHVs) from the local community who were recruited by the government were involved in the implementation of the study. Prior to data collection, 45 FCHVs, health workers and local leaders in the study area were informed about the study. They thereafter conveyed this information to the eligible women and invited them to participate in the study. One-day screening sites were prepared at health centers, local schools, and village halls.

### Data collection

A questionnaire was administered by trained female interviewers. Data on socio-demographic characteristics were collected, including age, ethnicity, religion, occupation, education level, household income and number of children. In addition, information was obtained on smoking habits (previous, current, or never smoking), frequency of intake of instant noodles, milk and vegetarian diet, and family history of diabetes, hypertension, and CVDs. Intake of instant noodles and milk was categorized as intake more often or less often than 2 times weekly. Data on energy intake and physical activity were not collected. The questionnaire was not validated.

### Measurement of anthropometric parameters and blood pressure

Height was measured in centimeters (cm) by a measuring tape attached on the wall. Participants were requested to remove any hair ties, take off their shoes, to stand on a flat surface and look straight ahead without tilting their head. Body weight was recorded in kilogram (kg) using a portable digital weighing scale. The participants were requested to remove their footwear and to have minimal clothing. WC was measured in a separate room by a female health worker, in standing position, at the end of a natural expiration, holding the arms relaxed at the sides and at the midpoint between the lower margin of the last palpable rib in the mid axillary line and the top of the iliac crest (hip bone). The measurement was recorded in centimeters. BMI was calculated as weight in kilogram per square of height in meter and categorized according to WHO cut-off values for Asians as underweight (< 18.5 kg/m^2^), normal weight (18.5–22.9 kg/m^2^), overweight (23.0–27.4 kg/m^2^) and obese (≥ 27.5 kg/m^2^) (27). For comparison with other studies, the prevalence of overweight/obesity was also calculated using cut-offs for the general population, overweight 25.0–29.9 kg/m^2^ and obesity ≥30 kg/m^2^. Central obesity was defined as WC ≥ 80 cm according to WHO’s cut-off for Asian and European women (27). Blood pressure (BP) was measured by a digital device (Omron-5 series digital BP monitor, Japan) on the left arm in sitting position at the end of the interview. Hypertension was defined as systolic BP ≥ 140 mmHg and/or diastolic BP ≥ 90 mmHg and/or self-report of hypertension. Prehypertension was defined as systolic BP 120–139 mmHg and/or diastolic BP 80–89 mmHg (33).

### Statistical analysis

The descriptive data are presented as mean and standard deviation (SD) for continuous variables and as frequencies (*n*) and percentages (%) for categorical variables. One-way ANOVA test was used for continuous variables and Pearson’s χ2 test for categorical variables. The overall prevalence of obesity across the covariates was calculated through cross tabulation. Bivariate and multivariate binary logistic regression analyses were performed with overweight/obesity (BMI ≥ 23 kg/m^2^) and central obesity (WC ≥ 80 cm) as dependent variables. Independent variables used in the analyses were: age, sociodemographic and dietary characteristics and smoking status. Age was used as a continuous variable or categorized into four groups, 17–34 years, 34–44 years, 45–54 years and ≥ 55 years. Ethnicity was classified into three groups, Brahmin/Chhetri, Adhivasi/Janajati and Dalit. Educational status was divided into two groups, uneducated and educated. Household monthly income was categorized into < and ≥ 24,000 Nepali rupees. Number of children was categorized as zero, 1–3 children and > 3 children. Intake of instant noodles and milk was categorized into <2 times a week and ≥ 2 times a week, respectively. Smoking status was categorized into current smoker, former smoker and never smoker. Adjustment for age, education and household income was made based on previous studies (12, 34). Results are presented as odds ratios (OR) and adjusted odds ratios (AOR) with 95% confidence intervals (CIs). All analyses were performed using IBM SPSS (version 28.0.1.0, New York, NY, USA).

### Ethical considerations

The study was approved by the Dhulikhel Hospital/Kathmandu University School of Medical Sciences Institutional Review Committee (Approval no. 38/2011), Nepal Health Research Council (Approval no. 124/2012), and the Regional Committee for Medical and Health Research Ethics, Central Norway (Ref. no 2011/2540). Informed consent in the form of a signature or thumb print was obtained from women who agreed to participate. The study was conducted according to the guidelines provided in the Declaration of Helsinki.

## Results

### Characteristics of the study population

Altogether, 1,391 women aged 17 to 86 years (43.1 ± 14.4 years) were finally included based on measurement of BMI. The characteristics of the study population are summarized in Table 1. The women belonged to three ethnic groups, Brahmin/Chhetri, Adhivasi/Janajati and Dalit, representing the advantaged cast, “middle class” and disadvantaged cast, respectively. The majority were part of the Adhivasi/Janajati ethnicity (69.5%). Most of the women were uneducated (85.8%), and agriculture was the main source of income (84.6%). Moreover, 556 (40.0%) reported any intake of instant noodles, and 22.0% reported intake ≥2 times weekly. Milk intake was reported by 819 (59.4%) participants and intake ≥2 times a week by 29.0% of the study participants. One fourth of the women (25.0%) were current smokers, and 7.0% were former smokers. Mean BMI and WC were 23.1 ± 4.6 kg/m^2^ and 77.1 ± 8.9 cm, respectively. Prehypertension and hypertension were observed in 506 (36.4%) and 184 (13.4%) women, respectively. Only 27 (2.0%) women reported that they had hypertension, however, we do not have data on medication.

**Table 1:** Characteristics of the study population (*n* = 1,391).

Characteristics	*n* (%) or mean ± SD
Age (years)	43.1 ± 14.4
**Age groups, years**	
17–34	401 (29.0)
35–44	401 (29.0)
45–54	305 (22.0)
≥ 55	284 (20.0)
**Ethnicity**	
Brahmin/Chhetri	170 (12.2)
Adhivasi/Janajati	967 (69.5)
Dalit	254 (18.3)
**Educational status**	
Uneducated	1,193 (85.8)
Educated	198 (14.2)
**Monthly household income (NPR) ^a^**	
< 24,000	510 (39.4)
≥ 24,000	785 (60.6)
**Number of children**	3.5 ± 1.9
Parity	
Null	46 (3.3)
1–3	685 (49.3)
> 3	660 (47.4)
Dietary factors	
**Vegetarian diet ^b^**	
Yes	102 (7.4)
No	1,277 (92.6)
**Instant noodle intake ^b^**	
< 2 times a week	1,073 (78.0)
≥ 2 times a week	306 (22.0)
**Milk intake ^b^**	
< 2 times a week	979 (71.0)
≥ 2 times a week	400 (29.0)
**Smoking status ^c^**	
Current	352 (25.0)
Former	93 (7.0)
Never	943 (68.0)
**Anthropometric measurements**	
Height (cm)	149.0 ± 6.8
Weight (kg)	51.2 ± 10.1
Body mass index (kg/m^2^)	23.1 ± 4.6
Waist circumference (cm) ^d^	77.1 ± 8.9
**Blood pressure (BP), mmHg^e^**	
Systolic BP	112.2 ± 15.7
Diastolic BP	72.5 ± 10.2

SD, standard deviation; NPR, Nepali rupee. ^a^ missing *n* = 96; ^b^ missing = 12; ^c^ missing *n* = 3; ^d^ missing *n* = 156, ^e^ missing *n* = 170.

### Prevalence of overweight/obesity and central obesity stratified by the participants’ characteristics

The prevalence of overweight (BMI 23.0–27.4 kg/m^2^) and obesity (BMI ≥ 27.5 kg/m^2^) was 30.5 and 12.0%, respectively (Table 2). When applying WHO’s BMI cut-offs for the general population, the prevalence of overweight (BMI 25.0–29.9 kg/m^2^) and obesity (BMI ≥ 30.0 kg/m^2^) was 17.0 and 6.7%, respectively. The prevalence of obesity was highest in the age groups 35–44 and 45–54 years, 14.4 and 12.4%, respectively. Underweight (BMI < 18.5 kg/m^2^) was observed in 9.6% of the study population, one third of these were in the age group 35–44 years. Hypertension was most frequent in the age group 45–54 years.

**Table 2:** Characteristics of the participants stratified by different BMI categories.

Characteristics	*n* (%) or mean ± SD	BMI cut-offs for Asians			
		Underweight	Normal weight	Overweight	Obesity
		< 18.5	18.5–22.9	23.0–27.4	≥ 27.5
Study population	1,391	134 (9.6)	666 (47.8)	424 (30.5)	167 (12.0)
Age (years), mean ± SD	43.1 ± 14.4	42.1 ± 13.9	42.9 ± 14.9	44.1 ± 14.2	42.4 ± 13.2
BMI (kg/m^2^), mean ± SD	23.1 ± 4.7	16.9 ± 1.5	20.9 ± 1.3	24.8 ± 1.3	32.3 ± 4.8
WC (cm) ^a^, mean ± SD	77.1 ± 8.9	71.4 ± 6.0	73.4 ± 6.5	79.8 ± 6.8	88.5 ± 9.9
**Age groups, (years)**					
17–34	401 (29.0)	36 (9.2)	201 (50.0)	120 (30.0)	44 (10.8)
35–44	401 (29.0)	45 (11.4)	188 (46.8)	110 (27.4)	58 (14.4)
45–54	305 (22.0)	31 (10.2)	137 (45.0)	99 (32.5)	38 (12.5)
≥ 55	284 (20.0)	22 (7.7)	140 (49.3)	95 (33.5)	27 (9.5)
**Ethnicity**					
Brahmin/Chhetri	170 (12.0)	15 (8.7)	73 (43.0)	62 (36.5)	20 (11.8)
Adhivasi/Janajati	967 (70.0)	88 (9.1)	466 (48.2)	295 (30.5)	118 (12.3)
Dalit	254 (18.0)	31 (12.2)	127 (50.0)	67 (26.4)	29 (11.4)
**Educational status**					
Uneducated	1,193 (85.7)	113 (9.5)	564 (47.3)	373 (31.2)	143 (11.9)
Educated	198 (14.3)	21 (10.6)	51.6 (102)	51 (25.8)	24 (12.0)
**Monthly household income in NPR ^b^**					
< 24,000	510 (39.4)	50 (9.8)	232 (45.5)	158 (31.0)	70 (13.7)
≥ 24,000	785 (60.6)	74 (9.4)	392 (50.0)	236 (30.0)	83 (10.6)
**Number of children**					
Null	46 (3.3)	5 (11.0)	19 (41.3)	14 (30.4)	8 (17.3)
1–3	685 (49.3)	64 (9.3)	332 (48.5)	208 (30.4)	81 (11.8)
> 3	660 (47.4)	65 (9.8)	315 (47.7)	202 (30.6)	78 (11.9)
Dietary factors					
Vegetarian diet ^c^					
Yes	102 (7.4)	5 (5.0)	44 (43.0)	37 (36.0)	16 (16.0)
No	1,277 (92.6)	128 (10.0)	618 (48.5)	381 (29.8)	150 (11.7)
**Instant noodle intake ^c^**					
< 2 times a week	1,073 (78.0)	105 (9.8)	528 (49.2)	317 (29.5)	23 (11.5)
≥ 2 times a week	306 (22.0)	28 (9.2)	134 (43.8)	101 (33.0)	43 (14.0)
**Milk intake ^c^**					
< 2 times a week	979 (71.0)	94 (9.6)	479 (48.9)	293 (30.0)	113 (11.5)
≥ 2 times a week	400 (29.0)	39 (9.8)	183 (45.8)	125 (31.2)	53 (13.2)
**Smoking status ^d^**					
Current	352 (25.3)	25 (7.1)	181 (51.4)	105 (29.8)	41 (11.6)
Former	93 (6.7)	11 (11.8)	46 (49.5)	22 (23.7)	14 (15.0)
Never	943 (68.0)	97 (10.3)	437 (46.2)	297 (31.5)	112 (12.0)
**Blood pressure, mmHg ^e^**					
Systolic BP	112.2 ± 15.7	110.3 ± 19.5	111.2 ± 15.6	112.9 ± 15.0	115.2 ± 16.2
Diastolic BP	72.5 ± 10.2	70.7 ± 10.5	71.5 ± 10.1	73.3 ± 10.2	75.5 ± 9.9

SD, standard deviation; BMI, body mass index; WC, waist circumference; NPR, Nepali rupee. ^a^ missing *n* = 158; ^b^ missing *n* = 96; ^c^ missing *n* = 12; ^d^ missing *n* = 3, ^e^ missing *n* = 170.

**Figure 1:**
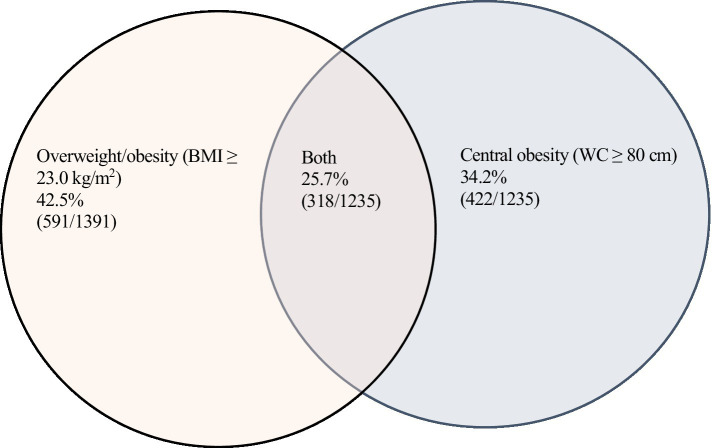
Venn diagram showing the prevalence of overweight/obesity, central obesity and the combination of both.

The prevalence of central obesity by selected characteristics is shown in Table 3. Central obesity (WC ≥ 80 cm) was observed in 34.2% of the participants and occurred frequently in all age groups. Women with intake of instant noodles ≥2 times per week displayed a higher prevalence of central obesity than those with intake <2 times weekly. Mean systolic and diastolic BP were higher in individuals with WC ≥ 80 cm than in those with WC < 80 cm. Figure 1 shows the percentage of the population with overweight/obesity, central obesity, and combination of the two. Altogether, 25.7% displayed both overweight/obesity and central obesity. Central obesity was also found in 14.9 and 15.3% of those with normal weight and underweight, respectively.

**Table 3:** Characteristics of the participants stratified by waist circumference below or above 80 cm.

Characteristics	Waist circumference categories		
	*n* (%)	< 80 cm	≥ 80 cm
Study population	1,235	813 (65.8)	422 (34.2)
Age (years), mean ± SD	43.0 ± 14.3	43.2 ± 14.3	42.7 ± 14.2
BMI (kg/m^2^), mean ± SD	23.1 ± 4.7	21.6 ± 3.4	26.0 ± 5.4
WC (cm), mean ± SD	77.1 ± 8.9	72.0 ± 4.4	87.0 ± 7.0
**Age groups, (years)**			
17–34	358 (29.0)	233 (65.0)	125 (35.0)
35–44	353 (28.6)	222 (63.0)	131 (37.0)
45–54	275 (22.4)	190 (69.0)	85 (31.0)
≥ 55	249 (20.0)	168 (67.5)	81 (32.5)
**Ethnicity**			
Brahmin/Chhetri	145 (11.7)	95 (65.5)	50 (34.5)
Adhivasi/Janajati	873 (70.7)	571 (65.4)	302 (34.6)
Dalit	217 (17.6)	147 (67.7)	70 (32.3)
**Educational status**			
Uneducated	1,061 (86.0)	698 (65.8)	363 (34.2)
Educated	174 (14.0)	115 (66.0)	59 (34.0)
**Monthly household income in NPR ^a^**			
< 24,000	435 (38.0)	273 (62.8)	162 (37.2)
≥ 24,000	712 (62.0)	478 (67.0)	234 (33.0)
**Number of children**			
Null	44 (3.6)	26 (59.0)	18 (41.0)
1–3	615 (49.8)	393 (64.0)	222 (36.0)
> 3	576 (46.6)	394 (68.4)	182 (31.6)
Dietary factors			
**Vegetarian ^b^**			
Yes	91 (7.4)	62 (68.0)	29 (32.0)
No	1,133 (92.6)	746 (65.8)	387 (34.2)
**Instant noodle intake ^b^**			
< 2 times a week	965 (79.0)	672 (69.6)	293 (30.4)
≥ 2 times a week	259 (21.0)	136 (52.5)	123 (47.5)
**Milk intake ^b^**			
< 2 times a week	878 (71.7)	611 (69.6)	267 (30.4)
≥ 2 times a week	346 (28.3)	197 (57.0)	149 (43.0)
**Smoking ^c^**			
Current	302 (24.5)	210 (69.5)	92 (30.5)
Former	82 (6.7)	53 (65.0)	29 (35.0)
Never	848 (68.8)	548 (65.0)	300 (35.0)
**Blood pressure, mmHg ^d^**			
Systolic BP	112.8 ± 15.6	111.5 ± 15.8	115.1 ± 15.2
Diastolic BP	72.5 ± 10.4	71.6 ± 10.3	74.3 ± 10.6

SD, standard deviation; BMI, body mass index; WC, waist circumference; NPR, Nepali rupees. ^a^ missing *n* = 88; ^b^ missing *n* = 11; ^c^ missing *n* = 3, ^d^ missing *n* = 306.

### Characteristics associated with overweight/obesity

Characteristics associated with overweight/obesity (cut-offs for Asians) are presented in Table 4. The prevalence of overweight/obesity did not convincingly differ by ethnicity. Women reporting consumption of instant noodles ≥2 times weekly exhibited a higher prevalence than those reporting intake <2 times weekly (OR 1.3, 95% CI: 0.99, 1.68; *p* = 0.062), however, borderline significant. Non-vegetarian diet was associated with a non-significant lower prevalence (OR 0.7, 95% CI: 0.47, 1.08; *p* = 0.112). Hypertension was associated with overweight/obesity (OR 1.5, 95% CI: 1.09, 2.13; *p* = 0.013).

**Table 4:** Characteristics associated with overweight/obesity (*n* = 1,257).

Characteristics	Normal weight (*n* %)	Overweight/obesity (*n* %)	OR (95% CI)	*p*-value	AOR (95% CI)	*p*-value
Age groups, years						
17–34	201 (55.0)	164 (45.0)	1		1	
35–44	188 (53.0)	168 (47.0)	1.1 (0.82, 1.47)	0.543	0.9 (0.68, 1.34)	0.792
45–54	137 (50.0)	137 (50.0)	1.2 (0.89, 1.68)	0.204	1.1 (0.75, 1.55)	0.665
≥ 55	140 (53.0)	122 (47.0)	1.1 (0.78, 1.47)	0.685	0.9 (0.62, 1.30)	0.570
**Ethnicity**						
Adhivasi/Janajati	466 (53.0)	413 (47.0)	1		1	
Brahmin/Chhetri	73 (47.0)	82 (53.0)	0.9 (0.63, 1.15)	0.293	0.9 (0.62, 1.18)	0.353
Dalit	127 (57.0)	96 (43.0)	1.3 (0.90, 1.78)	0.174	1.3 (0.88, 1.82)	0.195
**Educational Status**						
Educated	102 (57.6)	75 (42.4)	1		1	
Uneducated	564 (52.0)	516 (48.0)	1.2 (0.90, 1.75)	0.182	1.3 (0.86, 1.83)	0.232
**Household income per month ^a^, NPR**						
< 24,000	232 (50.4)	228 (49.4)	1		1	
≥ 24,000	392 (55.0)	319 (45.0)	0.8 (0.65, 1.05)	0.116	0.8 (0.66, 1.05)	0.124
**Number of children**						
1–3	332 (53.5)	289 (46.5)	1		1	
> 3	315 (53.0)	280 (47.0)	1.0 (0.81, 1.28)	0.856	0.9 (0.17, 1.21)	0.592
Null	19 (46.3)	22 (53.7)	1.3 (0.71, 2.51)	0.378	1.5 (0.80, 3.01)	0.197
**Vegetarian diet ^b^**						
Yes	44 (45.0)	53 (55.0)	1		1	
No	618 (54.0)	531 (46.0)	0.7 (0.47, 1.08)	0.112	1.0 (0.87, 1.01)	0.926
**Instant noodle intake ^b^**						
< 2 times per week	528 (54.5)	440 (45.5)	1		1	
≥ 2 times per week	134 (48.0)	144 (52.0)	1.3 (0.99, 1.68)	0.062	1.4 (1.03, 1.79)	0.032
**Milk ^b^**						
≥ 2 times per week	183 (51.0)	178 (49.0)	1		1	
< 2 times per week	479 (54.0)	406 (46.0)	0.9 (0.68, 1.11)	0.271	1.0 (0.87, 1.01)	0.715
**Smoking status ^c^**						
Never	437 (52.0)	409 (48.0)	1		1	
Current	181 (55.4)	146 (44.6)	0.9 (0.67, 1.11)	0.256	0.8 (0.62, 1.07)	0.146
Former	46 (56.0)	36 (44.0)	0.8 (0.53, 1.32)	0.442	0.7 (0.48, 1.26)	0.780
**Hypertension**						
No	592 (54.3)	497 (45.7)	1		1	
Yes	74 (44.0)	94 (56.0)	1.5 (1.09, 2.10)	0.013	1.4 (1.02, 2.04)	0.037

NPR, Nepalese rupee; OR, odds ratio; AOR, adjusted odds ratio for age, education, and household income. ^a^ missing *n* = 86, ^b^ missing *n* = 11, ^c^ missing *n* = 2.

### Characteristics associated with central obesity

Characteristics associated with central obesity are presented in Table 5. Women with more than 3 children tended to have a lower prevalence (OR 0.8, 95% CI: 0.64, 1.04; *p* = 0.131), however, not statistically significant. Women consuming instant noodles ≥2 times a week displayed a higher prevalence of central obesity compared to those with intake <2 times weekly, (OR 2.1, 95% CI: 1.57, 2.74; *p* < 0.001). Women with milk intake <2 times a week were less likely to have central obesity than those with intake ≥2 times a week, (OR 0.6, 95% CI: 0.45, 0.75; *p* < 0.001). Given that concomitant intake of instant noodles and milk was common, we regarded instant noodles as a potential confounder. Accordingly, when adjusting milk intake for instant noodle consumption, the association was attenuated (OR 0.8, 95% CI: 0.56, 1.03; *p* = 0.08). Central obesity was less prevalent in those with non-vegetarian diet and in current smokers (OR 0.7, 95% CI: 0.47, 1.08; *p* = 0.112) and (OR 0.8 95% CI: 0.60, 1.06; *p* = 0.122), respectively, however, not significant. Women with central obesity exhibited a higher prevalence of hypertension (OR 1.5, 95% CI: 1.11–2.13; *p* = 0.009).

**Table 5:** Characteristics associated with central obesity in the study population (*n* = 1,235).

Characteristics	Waist circumference		OR (95% CI)	*p*-value	AOR (95% CI)	*p*-value
Age groups, years	< 80 cm	≥ 80 cm				
≤ 34	233 (65.0)	125 (35.0)	1		1	
35–44	222 (63.0)	131 (37.0)	1.1 (0.81, 1.49)	0.542	1.0 (0.71,1.43)	0.964
45–54	190 (69.0)	85 (31.0)	0.8 (0.60, 1.17)	0.289	0.8 (0.54, 1.15)	0.216
≥ 55	168 (67.5)	81 (32.5)	0.9 (0.64, 1.27)	0.541	0.9 (0.60, 1.28)	0.486
**Ethnicity**						
Adhivasi/Janajati	571 (65.4)	302 (34.6)	1		1	
Brahmin/Chhetri	95 (65.5)	50 (34.5)	1.0 (0.69, 1.44)	0.976	1.1 (0.74, 1.62)	0.641
Dalit	147 (68.0)	70 (32.0)	0.9 (0.66, 1.27)	0.516	0.9 (0.64, 1.26)	0.543
**Educational status**						
Educated	115 (66.0)	59 (34.0)	1		1	
Uneducated	698 (65.8)	363 (34.2)	1.0 (0.72, 1.38)	0.937	1.1 (0.72, 1.58)	0.740
**Household income per month ^a^, NPR**						
< 24,000	273 (63.0)	162 (37.0)	1		1	
≥ 24,000	478 (67.0)	234 (33.0)	0.8 (0.64, 1.06)	0.131	0.8 (0.35, 1.05)	0.816
**Number of children**						
1/3	393 (64.0)	222 (36.0)	1		1	
> 3	394 (68.4)	182 (31.6)	0.8 (0.64, 1.04)	0.101	0.8 (0.60, 1.06)	0.799
Null	26 (59.0)	18 (41.0)	1.2 (0.66, 2.28)	0.522	1.3 (0.71, 2.58)	0.364
**Vegetarian diet ^b^**						
Yes	62 (68.0)	29 (32.0)	1			
No	746 (66.0)	387 (34.0)	1.1 (0.70, 1.75)	0.658	1.0 (0.58, 1.63)	0.926
**Instant noodle intake ^b^**						
< 2 times per week	672 (69.6)	293 (30.4)	1		1	
≥ 2 times per week	136 (52.5)	123 (47.5)	2.1 (1.57, 2.74)	< 0.001	2.2 (1.67, 2.99)	< 0.001
**Milk intake ^b^**						
≥ 2 times per week	197 (57.0)	149 (43.0)	1		1	
< 2 times per week	611 (70.0)	267 (30.0)	0.6 (0.45, 0.75)	< 0.001	0.5 (0.40, 0.69)	< 0.001
**Smoking status ^c^**						
Never	548 (64.6)	300 (35.4)	1		1	
Current	210 (69.5)	92 (30.5)	0.8 (0.60, 1.06)	0.122	0.8 (0.60, 1.10)	0.175
Former	53 (64.6)	29 (35.4)	1.0 (0.62, 1.61)	0.998	1.1 (0.67, 1.81)	0.690
**Hypertension**						
No	713 (67.0)	347 (33.0)	1		1	
Yes	100 (57.0)	75 (43.0)	1.5 (1.11, 2.13)	0.009	1.6 (1.14, 2.27)	0.006

NPR, Nepalese rupee; OR, odds ratio; AOR, adjusted odds ratio for age, education, and household income. ^a^ missing *n* = 88; ^b^ missing *n* = 11; ^c^ missing *n* = 3.

## Discussion

In this large, cross-sectional study we observed a high prevalence of overweight/obesity, as well as central obesity among women in a rural district of Nepal. We applied cut-offs for Asians as recommended by WHO, both for BMI and WC. The prevalence of overweight and obesity in the study population was 30.5 and 12.0%, respectively, whereas central obesity was observed in 34.2%. Moreover, 9.8% of the women were underweight. To our knowledge, this is the first report on assessment of both overweight/obesity and central obesity among women specifically in rural Nepal. This approach enabled identification of central obesity among subjects in different BMI categories. Central obesity was observed in 15.3, 14.9 and 25.7% of those with underweight, normal body weight and overweight/obesity, respectively. Hypertension was observed in 13.4% and was associated with both overweight/obesity and central obesity. Women reporting intake of instant noodles more than two times weekly had a higher prevalence of central obesity.

The high prevalence of overweight and obesity observed among women in rural Nepal complies with the alarmingly high prevalence worldwide. Women are more susceptible to be overweight/obese, both in high-income and low-income countries (5, 25). We observed a higher prevalence of overweight/obesity than reported by Rawal et al., among women from the NDHS 2016, using cut-offs for Asians, 42.5% versus 32.9% (8). Mothers in a semi-urban region of Nepal displayed a prevalence of 57% (≥ 23.0 kg/m^2^) (35). The majority of the participants in the NDHS resided in urban areas (64.6%), where the prevalence of overweight and obesity is anticipated to be higher than in rural districts (11). However, Rawal et al., observed no significant difference in prevalence of overweight/obesity by ecological regions and place of residence (urban vs. rural) (8). In the NDHS, women in the age 36–45 years were more likely to be overweight or obese (8).

BMI is easy to obtain and is the most widely used measure of obesity and of the association between obesity and morbidity/mortality. However, it has been questioned whether BMI is an appropriate indicator of obesity (36). BMI does not distinguish between fat and lean mass and does not give information about fat accumulation (37). A novelty of the present study is that we applied both BMI and WC to assess the prevalence of overweight/obesity and central obesity, respectively, among women specifically in a rural district of Nepal. We used a WC cut-off of ≥80 cm as recommended by WHO for Asian and European women and found that 34.2% of the women displayed central obesity. The highest prevalence was seen in the age group 35–44 years (37%), followed by women <34 years. In a secondary analysis of NCDs risk factor 2013, addressing metabolic syndrome in 3,729 Nepalese adults, 27.5% had high WC (38). When stratifying for sex, central obesity was observed in 36.5% of the women (38), which is in line with the prevalence in our study. A report from “Non-communicable disease risk factors: STEPS survey Nepal 2019” showed a somewhat higher prevalence among the women, 39.7% versus 34.2% in the present study (39). Both studies applied cut-offs for Asians to measure WC (38, 39).

The high prevalence of overweight/obesity and central obesity in our study population is of concern, as these conditions are associated with increased risk of NCDs like T2D, hypertension, CVDs and cancer, giving rise to excess morbidity and mortality (16, 40, 41). High BMI has been associated with a wide range of cancers, and the combination of high BMI and WC with all-cause cancer (40, 41). Notably, a fourth of the women in the present study had both overweight/obesity and central obesity. Moreover, 14.9 and 15.3% of those with normal weight and underweight, respectively, displayed central obesity. There is increasing evidence that individuals with normal-weight central obesity are at increased risk for T2D due to excessive accumulation of abdominal fat (36, 42). Furthermore, subjects with central obesity at normal BMI have been shown to exhibit a similar or higher mortality risk as those with central obesity who are overweight or obese (43). They have, however, received little clinical attention, and are overlooked when it comes to development of preventive strategies. There are no reports on the significance of the combination of underweight and central obesity, but it is reasonable that these individuals have a similar risk profile as normal-weight subjects with central obesity. The American Obesity Society recommends routine measurement of WC in overweight people, but not in subjects with normal weight as the evidence of harmful effects of central obesity in individuals with a normal BMI is considered to be limited (44). Given the rise in central obesity over the last decades, the proportion of individuals with normal-weight and underweight central obesity is anticipated to rise. Accordingly, identification of these individuals and assessment of their health risks are clinically important.

Underweight was also prevalent in our study population, affecting 9.8% of the women, but was lower than reported by Rawal et al., from the NDHS 2016, where a prevalence of 18% was observed among women (8). This could possibly reflect differences between urban and rural districts. In the present study, underweight was most common in the age group 35–44 years, whereas Rawal et al., observed that older adult (≥ 65 years), and adults of the poorest wealth quintile were more likely to be underweight (8).

The high prevalence of overweight/obesity and central obesity as observed in the present and previous studies is postulated to be attributed to the rapid urbanization and industrialization. This has caused a dramatic shift in dietary patterns from traditional to western diet with energy-dense foods, accompanied by a decline in physical activity levels. The Nepalese diet has been reported to change from agricultural staple-based foods to modern processed foods with higher total energy, total fat, and sugar. The most pronounced increase was observed in consumption of plant oils with a seven-fold rise from 10 g/capita/day in 1970 to 65 g/capita/day in 2010 (45). Instant noodles were introduced in Nepal about 30 years ago. In 2019, South Korea had the highest consumption of instant noodles per capita with 75 servings per year, followed by Nepal and Indonesia with 58 and 56 servings, respectively (46). Notably, instant noodles have a high concentration of refined carbohydrates, fats and sodium and a high calorie content (47), and may thus contribute to an increased risk of metabolic disease. In a recent study, Park et al. showed that noodle intake had a causal association with metabolic syndrome in Asian adults (48). Moreover, they found that individuals in the high-noodle intake group had lower intake of calcium, vitamin D, and flavonoids, indicating a poorer diet quality. Additionally, the glycemic index and glycemic load of their daily meals were significantly higher compared to those in the low-noodle intake group (48). A South Korean study including 10,711 subjects, 19–64 years of age, also showed that women who consumed instant noodles ≥2 times a week, were 68% more likely to develop metabolic syndrome (49). These studies comply with our findings showing that intake of instant noodles ≥2 times a week was associated with central obesity and overweight/obesity, although borderline significant for the latter.

Noticeably, milk intake more than twice weekly was associated with increased prevalence of central obesity in our study population. This contrasts with the majority of studies showing a protective effect of dairy products both against central obesity and overweight/obesity (50, 51). The milk consumed in rural Nepal is not reduced in fat content, however, based on previous studies, this is not associated with an increased prevalence of obesity (52, 53). The association could possibly be attributed to the fact that a large proportion of women reporting milk intake ≥2 times weekly also consumed instant noodles. When adjusting milk intake ≥2 times weekly for intake of instant noodles twice weekly, the association was attenuated.

A decrease in physical activity has been recognized as an important contributor to obesity and NCDs. A nationwide cross-sectional study among 4,143 Nepalese adults (66.5% females) aged 15–69 years showed that around 97% of men and 98% of women met the recommended levels of physical activity. The Global Physical Activity Questionnaire (GPAQ) was applied and both rural and urban populations were included. Very few were engaged in any leisure-time activity, whereas both men and women reported high occupational physical activity. Moreover, a multiple regression analysis showed that less self-reported total physical was inversely associated with older age, higher level of education, urban place of residence, never been married, being underweight, and smoking in both sexes, and with overweight and obesity in males (54). Unfortunately, we did not collect data on physical activity, however, given that the majority of our study subjects were farmers, it is reasonable that the pattern of physical activity was similar.

In the NDHS 2016, adults who had no education except for preschool, had reduced prevalence of overweight/obesity (8), which contrasts with our results. Adults who never married also displayed reduced risk of overweight/obesity (8). We did not have the possibility to explore whether married women displayed a higher prevalence than never married, as only married women were included. Most studies show that increasing number of children is associated with obesity (55). Weng et al., observed a 7% increase in risk of obesity among women for each additional child after adjustment for multiple factors (56). In the present study, those with more than three children tended to have a lower prevalence of central obesity, whereas no association with BMI-defined obesity was observed.

There is emerging evidence for a role of environmental pollution in the development of obesity (57). These pollutants are referred to as obesogens, and they include among others bisphenol A (BPA), which is added to plastics and widely used, as well as some pesticides and air pollution (58–60). Notably, in 2019 Nepal was ranked as number 2 among countries with the highest emission of outdoor particulate matter 2.5 (PM_2.5_), which is the major air pollution globally (61). Kathmandu has topped the global ranks of the most polluted city for the last few years (62). We do not have data on air pollution at our study site in 2012–13. The study site is, however, located not so far from Kathmandu.

In the present study, overweight/obesity and central obesity were frequent in all age groups, thus affecting a large proportion of women in fertile age. Both women who are underweight and overweight/obese are at risk for adverse pregnancy outcomes, among others maternal anemia, gestational diabetes, miscarriage, preterm deliveries, intrauterine growth retardation and low birth weight (63–65). Furthermore, the offspring’s future health may be affected negatively, as children born to obese mothers are at increased risk of obesity and T2D (20, 66). This may be attributed to epigenetic mechanisms, genetic predisposition, or a shared family environment (20, 66). This may contribute to acceleration of the obesity and diabetes epidemics in Asia. Our study underscores the need for urgent preventive and curative strategies to combat these epidemics.

The high prevalence of both communicable diseases and NCDs in Nepal is a significant public health concern and poses a substantial burden on the society. The Multisectoral Action Plan for Prevention and Control of NCDs 2014–2020 was formulated with the goal to reduce preventable morbidity, avoidable disability, and premature mortality due to *NCDs* in Nepal (67). In a recent report by Dhimal et al., it was stated that implementation of this plan has been challenging, with limited participation from non-health sectors (68). It was concluded that multisectoral action plans beyond 2020, should engage stakeholders from federal, provincial, and local governments and develop costed action plans with specific roles and responsibilities for each sector.

A strength of the present study is the large study sample and the acceptable participation rate of 62% (31). Moreover, assessment of both BMI and WC allows identification of individuals that may have increased cardiometabolic risk. The study has some limitations. Due to the cross-sectional design of the study, a cause-and-effect relationship cannot be established. We do not have data on the women who did not participate, and cannot exclude that these women differed from those who took part in the study. Thus, selection bias at that level cannot be ruled out. The findings of the present study may not be generalizable to women in all rural districts of Nepal or to men. Data on the intake of different food items were based on reported frequency and not amount. Moreover, the information collected from the questionnaire relies on self-report and may be influenced by recall and social desirability bias. Finally, the questionnaire was not validated.

## Conclusion

In conclusion, we observed a high prevalence of overweight/obesity and central obesity among women in a rural district of Nepal. The prevalence was high in all age groups. Central obesity was also found among women with normal weight and underweight. Intake of instant noodles ≥2 times weekly was associated with a higher prevalence of overweight/obesity and central obesity. Our results underscore that measurement of WC should be implemented in addition to BMI in assessment of obesity. Moreover, our findings illustrate the need for increased awareness and development of preventive health strategies to combat the obesity epidemic.

## Data Availability

The raw data supporting the conclusions of this article will be made available by the authors, without undue reservation.
